# The relationship between oral health status, loneliness, and sleep quality among the migrant elderly following children in Weifang, Shandong Province, China: A comparative analysis on different migration types

**DOI:** 10.3389/fpubh.2022.1053690

**Published:** 2023-02-08

**Authors:** Xiaoxu Jiang, Guangwen Liu, Jing Xu, Hexian Li, Jieru Wang, Mingli Pang, Shixue Li, Lingzhong Xu, Xiaolei Guo, Fanlei Kong

**Affiliations:** ^1^Centre for Health Management and Policy Research, School of Public Health, Cheeloo College of Medicine, Shandong University, Jinan, China; ^2^NHC Key Lab of Health Economics and Policy Research, Shandong University, Jinan, China; ^3^Shandong Center for Disease Control and Prevention, Academy of Preventive Medicine, Shandong University, Jinan, China

**Keywords:** oral health status, loneliness, sleep quality, migrant elderly following children, migration type disparity, structural equation modeling

## Abstract

**Background:**

The migrant elderly following children (MEFC) are a vulnerable group that emerged during fast urbanization in China. The MEFC faced physical and psychological discomfort upon their arrival in the inflow city, particularly those who came from rural areas.

**Objective:**

This study aimed to explore the relationship between oral health status, loneliness, and sleep quality among the MEFC in China and to clarify the disparities in the above mentioned relationship by migration type.

**Methods:**

In 2021, a cross-sectional survey was conducted in Weifang, Shandong Province, using multistage cluster random sampling to collect data from the MEFC aged 60 years and over. In total, 613 respondents [525 rural-to-urban (RTU) and 88 urban-to-urban (UTU)] were included in the final database. The chi-square test, *t*-test, and structural equation modeling (SEM) were used to investigate the relationship between oral health status, loneliness, and sleep quality among the RTU and UTU MEFC.

**Results:**

Total scores [mean ± standard deviation (SD)] for oral health status, loneliness, and sleep quality were 54.95 ± 6.47, 8.58 ± 3.03, and 4.47 ± 3.60, respectively. SEM revealed that, among the RTU and UTU MEFC, oral health status was positively and significantly related to sleep quality; however, the correlation was slightly stronger in the UTU MEFC. In both groups, there was a significant negative correlation between oral health status and loneliness, which was stronger in the UTU MEFC. In the RTU MEFC, a significant negative correlation between loneliness and sleep quality was observed, and in the UTU MEFC, no significant association between loneliness and sleep quality was observed.

**Conclusion:**

The sleep quality among the MEFC in this study was higher compared to previous studies. Oral health status was negatively correlated with loneliness and positively associated with sleep quality, whereas loneliness was negatively correlated with sleep quality. These three associations differed significantly between the UTU and RTU MEFC. The government, society, and families should take measures to improve oral health and reduce loneliness among the MEFC to improve their sleep quality.

## 1. Introduction

The aging population is rapidly growing worldwide (1). Meanwhile, population aging is much faster in China than in other developing countries, with the proportion of people aged 60 years and above increasing from 12.4% in 2010 to 28% in 2040 (2). Urbanization in China has also accelerated over the past few decades, resulting in the highest number of domestic migrants worldwide (376 million) in 2021 (3, 4). The migrant elderly following children (MEFC) are a vulnerable group that emerged during rapid urbanization in China. They are the elderly who migrate with their children to urban cities to take care of their grandchildren (5). Geographical mobility could affect the health of the migrant elderly (6); therefore, the MEFC face physical and psychological discomfort upon their arrival in the inflow city (7), especially those who migrated from rural areas (8, 9). Therefore, it is vital to conduct research on the MEFC groups to achieve healthy aging in China (10).

Previous studies confirmed an empirical relationship between oral health and sleep quality. For instance, a cross-sectional study in Brazil uncovered a negative association between the number of teeth and sleep quality (11). Al-Zahrani et al. (12) observed that the loss of a posterior tooth could cause bite imbalances and that tooth pain and discomfort could negatively affect sleep quality and duration. In a Japanese study, older people with <10 teeth were more likely to experience sleep disorders than those with ≥20 teeth (13). Therefore, it is necessary to study the mental and physical health of the elderly (14).

Loneliness is a common phenomenon among older people and has become a major public health challenge (15). Social distancing during the pandemic exacerbated loneliness among older adults (16). Few studies attempted to clarify the link between loneliness and sleep quality. A British study found that loneliness contributed to sleep disturbances and increased morbidity and mortality among older people (17). An Israeli study confirmed that loneliness was related to more sleep challenges: a stronger feeling of loneliness correlated with worse sleep quality (18). A longitudinal study found that loneliness represented a weaker social network and worse mood, causing the brain to secrete substances, i.e., cortisol, which could further affect sleep duration and quality (19).

Previous studies also indicated that oral diseases in older people may cause mental impairment (20), such as cognitive decline (21), emotional challenges (22), and reduced daily activities (23). Rouxel et al. noted that oral health was an individual risk factor for loneliness in older people, including oral diseases and tooth losses, which significantly influences the quality of life and wellbeing among older people (15). Walther et al.'s study uncovered that loneliness increased when self-rated health decreased, and dental appointments were postponed due to costs among older women (24). Qi et al.'s study of the Chinese elderly indicated that the remaining number of teeth and the rate of tooth loss were closely related to social isolation and loneliness (25). Therefore, the aging population requires more attention due to their physical fragility (26).

Previous studies have explored the relationship between (1) oral health and sleep quality, (2) oral health and loneliness, (3) loneliness and sleep quality separately. However, no study has investigated the simultaneous association between oral health status, loneliness, and sleep quality among the MEFC. Thus, this study aimed to assess the abovementioned relationships and further investigate the migration type disparity among the MEFC in Weifang, Shandong Province to provide evidence-based policy to improve the health status among the MEFC.

## 2. Materials and methods

### 2.1. Study location

The data for this study were collected from Weifang, Shandong Province, China in August 2021. Weifang, located in the center of the Shandong Peninsula, is a prefecture-level city in Shandong Province, known as the capital of kites. Weifang has 12 districts, and its gross domestic product (GDP) would increase by 9.70% over the previous year to RMB ¥701.06 billion (USD $103.76 billion) in 2021 (27). In 2020, the migrant population in Weifang would be 237.55 million, an increase of 112.17% from 2010 (28).

### 2.2. Data collection and research participants

For the selection of study subjects, multistage cluster random sampling was used. In the first stage of data collection, four of the 12 districts were chosen as primary sampling units (PSUs) based on their economic development and geographical region. In the second stage, four subdistricts were chosen as secondary sampling units (SSUs) from each of the PSUs. In the third stage, four communities from each SSU were chosen. The total sample for this study included all migrants over the age of 60 who had followed their children to Weifang in these four communities. The inclusion criteria for the respondents were: (1) participants >60 years; (2) participants who followed their children to Weifang; (3) participants with communication ability; and (4) participants who participated voluntarily and signed informed consent.

In total, 25 university students were trained as investigators based on their background knowledge, questionnaire content, and social survey methods during the survey. Participants and researchers participated in in-person interviews for 30 min to gather data. In total, 616 questionnaires were collected. However, 613 older adults were eventually included due to significant logical errors or incomplete questions in all three questionnaires.

### 2.3. Ethical approval

The survey and data were obtained with informed consent from all participants. The Ethics Committee of Shandong University reviewed and approved this study (No. 20180225).

### 2.4. Measurements

#### 2.4.1. Sociodemographic characteristics

Sociodemographic information included sex, age, education level, marital status, pension, and migration years and space range. Educational level was classified as primary school and below; junior high school; high school or technical secondary school; and a university degree or above. In this study, marital status was determined by the presence or absence of a spouse. Respondents were asked whether they received a pension. Years of migration was subdivided into more than 5 years and <5 years. Regarding the migration space range, there were three options: cross-district/county, cross-prefecture-level cities, and cross-provincial.

#### 2.4.2. Oral health status

Oral health status is measured by the Geriatric Oral Health Assessment Index (GOHAI). The GOHAI is a self-report instrument that measures the oral health status among older adults. It focuses on oral health function and psychosocial impact and has three dimensions: physical function, psychosocial function, and pain and discomfort. The GOHAI is determined using a five-point Likert scale: 1 = always, 2 = often, 3 = sometimes, 4 = rarely, and 5 = never. The total score of GOHAI is 12–60, and the higher the score, the better the oral health status. Wang et al. demonstrated the reliability and validity of GOHAI with excellent internal consistency in elderly people in mainland China (29).

#### 2.4.3. Loneliness

Loneliness was assessed using the UCLA (University of California Los Angeles) Loneliness Scale. The scale measures the emotions of loneliness caused by the discrepancy between the desired level of social engagement and the one that occurs. The full scale has 20 items, the options are on a Likert-type scale: l = never, 2 = rarely, 3 = sometimes, and 4 = always. This study uses the ULS-6, which is a simplified version of the UCLA Loneliness Scale and includes the following six items: often feel a lack of friends, often feel no one could be trusted, often feel left out, often feel separated from others, often feel shy, and often feel surrounded by people but not cared for. It can be concluded that the ULS-6 has shown good reliability in evaluating loneliness in Chinese older adults (30). In this study, Cronbach's alpha of the ULS-6 is 0.82, Kaiser–Meyer–Olkin (KMO) coefficient is 0.84, and Bartlett's sphericity test has a *p* < 0.05, indicating that the scale has good reliability and validity.

#### 2.4.4. Sleep quality

The Pittsburgh Sleep Quality Index (PSQI) assessed subjective sleep quality in the previous month. It comprises 19 self-rated and five other-rated items, totaling seven components scored on a scale of 0–3. The total PSQI score is the sum of the scores for each component and ranges from 0 to 21, with higher scores indicating worse sleep quality. In the elderly Chinese population, the PSQI has high reliability and validity (31). The seven components are subjective sleep quality, latency, continuity, habitual efficiency, disorder, use of sleep medication, and daytime dysfunction.

### 2.5. Statistical analysis

The sociodemographic characteristics of all participants in this study were characterized using descriptive statistics. The migration disparity in the sociodemographic characteristics of the MEFC was determined using the chi-square test. The migration disparity in oral health status, loneliness, and sleep quality was determined using the *t*-test. Statistical significance was set at 0.05, and all analyses were carried out using the Statistical Package for Social Sciences version 26.0 (SPSS, IBM, Armonk, New York, USA). Structural equation modeling (SEM) was used to investigate the impact of migration type on the association between oral health status, loneliness, and sleep quality among the MEFC in Weifang, Shandong Province. The best fit model was estimated using the maximum likelihood estimate. This study used fitness indices; goodness-of-fit index (GFI), adjusted goodness-of-fit index (AGFI), comparative fit index (CFI), and root mean square error of approximation (RMSEA). Hypothetical models were considered well-fitted to the data when GFI was >0.90, AGFI was >0.90, CFI was >0.90, and RMSEA was <0.05 (32). SEM was run using AMOS version 24.0 (IBM, Armonk, New York, NY, USA).

## 3. Results

### 3.1. Sample characteristics

Table 1 presents the demographic characteristics of the sample, with 613 MEFC included in the data analysis; 525 from rural areas and 88 from urban areas, indicating that the MEFC in this study were mainly from rural areas. Among the rural-to-urban (RTU) MEFC, 400 MEFC were women (76.2%), revealing that most RTU MEFC were elderly women; 496 were aged between 60 and 75 years (94.5%); most had a low educational level, 322 had only a primary school diploma or less (61.3%), and only two had a university education (0.4%); 459 had a spouse (87.4%); 234 received a pension (44.6%), confirming the low pension coverage for the rural elderly; 303 had moved in <5 years (57.7%), indicating that the main purpose of their migration was to take care of their grandchildren; 381 people moved across districts and counties (72.6%), 114 across prefecture-level cities (21.7%), and only 30 across provinces (5.7%), suggesting that most RTU MEFC migration distances were not too far. Concerning the urban-to-urban (UTU) MEFC, 48 were women (54.5%); 72 were aged between 60 and 75 years (81.8%); 24 had primary school education level or below (27.3%), 16 had a university degree (18.2%), revealing that most UTU MEFC were well educated; 80 had a spouse (90.9%); 80 received a pension (90.9%), indicating that the urban elderly had a wider pension coverage; 43 had moved in <5 years (48.9%); 49 moved across districts and counties (55.7%), 16 moved across prefecture-level cities (18.2%), and 23 were across provinces (26.1%).

**Table 1:** Characteristics of the migrant elderly following children (MEFC) by migration type.

**Variables**	**Total**	**Migration type**		**χ^2^/*P***
	**(*****n*** = **613)**	**RTU (*****n*** = **525)**	**UTU (*****n*** = **88)**	
**Gender**				
Male	165 (26.9)	125 (23.8)	40 (45.5)	17.950^***^
Female	448 (73.1)	400 (76.2)	48 (54.5)	
**Age**				
60–75	568 (92.7)	496 (94.5)	72 (81.8)	17.753^***^
76 years old and above	45 (7.3)	29 (5.5)	16 (18.2)	
**Education level**				
Primary school and below	346 (56.4)	322 (61.3)	24 (27.3)	109.561^***^
Junior high school	158 (25.8)	135 (25.7)	23 (26.1)	
High school/technical secondary school	91 (14.8)	66 (12.6)	25 (28.4)	
University degree or above	18 (3.0)	2 (0.4)	16 (18.2)	
**Marital status**				
With a spouse	539 (87.9)	459 (87.4)	80 (90.9)	0.860
No spouse	74 (12.1)	66 (12.6)	8 (9.1)	
**Having a pension**				
Yes	314 (51.2)	234 (44.6)	80 (90.9)	64.769^***^
No	299 (48.8)	291 (55.4)	8 (9.1)	
**Years of migration**				
5 years and below	346 (56.4)	303 (57.7)	43 (48.9)	2.401
More than 5 years	267 (43.6)	222 (42.3)	45 (51.1)	
Migration space range				
Cross-district/county	430 (70.1)	381 (72.6)	49 (55.7)	39.863^***^
Cross prefecture level cities	130 (21.2)	114 (21.7)	16 (18.2)	
Cross-provincial	53 (8.7)	30 (5.7)	23 (26.1)	

RTU, rural-to-urban; UTU, urban-to-urban.

*** p < 0.001.

The differences between the RTU and UTU MEFC were significant in sex, age, education level, pension, and migration space range. Specifically, the RTU MEFC had more women than the UTU MEFC, and pension coverage was much lower in the RTU MEFC than in the UTU MEFC. Additionally, there were more people over the age of 75 in the UTU MEFC than in the RTU MEFC, and a higher percentage of people with higher education in the UTU MEFC than in the RTU MEFC. Additionally, more people moved across provinces compared to the RTU MEFC.

Table 2 presents the general characteristics of oral health status, loneliness, and sleep quality according to migration type. The total GOHAI score was 54.65 ± 6.69 for the RTU MEFC and 56.72 ± 4.62 for the UTU MEFC, suggesting that the GOHAI scores were higher in the latter. Statistical differences were observed between the RTU and UTU MEFC in the total GOHAI score (*t* = −2.786, *p* < 0.001), physical function (*t* = −2.468, *p* < 0.01), psychosocial function (*t* = −1.975, *p* < 0.01), and pain and discomfort (*t* = −2.568, *p* < 0.01), indicating that the RTU MEFC obtained lower GOHAI scores than the UTU MEFC in all three aspects.

**Table 2:** General characteristics of the Geriatric Oral Health Assessment Index (GOHAI), loneliness, and sleep quality among the participants by migration type [mean ± standard deviation (SD)].

**Variables**	**Total**	**Migration type**		**t/*P***
	**(*****n*** = **613)**	**RTU (*****n*** = **525)**	**UTU (*****n*** = **88)**	
**GOHAI**				
Total	54.95 ± 6.47	54.65 ± 6.69	56.72 ± 4.62	2.786^***^
Physical function	17.35 ± 3.44	17.21 ± 3.49	18.18 ± 3.03	−2.468^**^
Psychosocial function	24.10 ± 2.06	24.03 ± 2.15	24.50 ± 1.35	1.975^***^
Pain and discomfort	13.50 ± 2.12	13.41 ± 2.14	14.03 ± 1.86	−2.568^**^
**Loneliness (ULS-6)**				
Total	8.58 ± 3.03	8.63 ± 3.06	8.27 ± 2.89	1.019
Often feel lack of friends	1.53 ± 0.79	1.55 ± 0.79	1.40 ± 0.75	1.641^*^
Often feel no one can be trusted	1.47 ± 0.73	1.47 ± 0.74	1.47 ± 0.69	0.032
Often feel left out	1.37 ± 0.62	1.38 ± 0.63	1.28 ± 0.55	1.354^*^
Often feel separated from others	1.37 ± 0.67	1.37 ± 0.67	1.38 ± 0.67	−0.071
Often feel shy	1.36 ± 0.64	1.37 ± 0.64	1.32 ± 0.62	0.675
Often feel surrounded by people but not cared	1.49 ± 0.72	1.50 ± 0.73	1.43 ± 0.64	0.765
**Sleep quality (PSQI)**				
Total	4.47 ± 3.60	4.57 ± 3.57	3.88 ± 3.34	1.197
Subjective sleep quality	0.88 ± 0.83	0.89 ± 0.82	0.80 ± 0.83	0.100
Sleep latency	1.11 ± 1.20	1.15 ± 1.21	0.92 ± 1.14	4.546^*^
Sleep Continuity	0.84 ± 0.91	0.84 ± 0.90	0.85 ± 0.94	0.629
Habitual sleep efficiency	0.16 ± 0.50	0.17 ± 0.51	0.11 ± 0.49	2.948
Sleep disorder	0.97 ± 0.56	0.99 ± 0.56	0.84 ± 0.52	2.003
Use of sleep medicine	0.13 ± 0.55	0.14 ± 0.57	0.06 ± 0.38	7.055^**^
Daytime dysfunction	0.52 ± 0.78	0.53 ± 0.78	0.43 ± 0.75	1.452

RTU, rural-to-urban; UTU, urban-to-urban.

* p < 0.05,

** p < 0.01,

*** p < 0.001.

The total loneliness score for the RTU and UTU MEFC was 8.63 ± 3.06 and 8.27 ± 2.89, respectively, with no significant differences (*t* = 1.019, *p* = 0.353). There were significant differences between the two items: often feeling without friends (*t* =1.641, *p* < 0.05) and feeling left out (*t* =1.354, *p* < 0.05), implying that the RTU MEFC felt more without friends and left out.

No statistical significance was observed in the total sleep quality score between the RTU (4.57 ± 3.57) and UTU (3.88 ± 3.34) MEFC. However, there were significant differences between sleep latency (*t* = 4.546, *p* < 0.05) and the use of sleep medications (*t* = 7.055, *p* < 0.05), indicating that the RTU MEFC had poor sleep quality.

### 3.2. The structural model

#### 3.2.1. Measurement consistency across migration types

The related fit statistics of the invariance of measures across migration type and fitness index for the five chosen models are presented in Table 3. First, the fitness indices of the RTU and UTU MEFC were compared to determine whether the variable “migration type” was suitable for group comparison.

**Table 3:** Multi-group model invariance test.

**Model**	**χ^2^**	**df**	**χ^2^/df**	**GFI**	**AGFI**	**CFI**	**RMSEA**	**ΔCFI**	**ΔRMSEA**
M_1_	410.613^***^	200	2.053	0.924	0.897	0.929	0.042	–	–
M_2_	410.613^***^	200	2.053	0.924	0.897	0.929	0.042	0	0
M_3_	410.613^***^	200	2.053	0.924	0.897	0.929	0.042	0	0
M_4_	428.586^***^	213	2.012	0.921	0.900	0.928	0.041	0.001	0.001
M_5_	437.204^***^	216	2.024	0.919	0.898	0.926	0.041	0.002	0

M_1_, RTU MEFC; M_2_, UTU MEFC; M_3_, unconstrained; M_4_, measurement weights; M_5_, structural weights. χ^2^, Chi-square; df, degrees of freedom; GFI, goodness-of-fit index; AGFI, adjusted goodness-of-fit index; CFI, comparative fitness index; RMSEA, root mean square error of approximation; ΔCFI, change of CFI; ΔRMSEA, change of RMSEA.

*** p < 0.001.

The GFI, AGFI, CFI, and RMSEA were the model fitness indices used in this study. The fitness indices of the RTU and UTU MEFC were identical in M_1_ and M_2_ (GFI = 0.924, AGFI = 0.897, CFI = 0.929, and RMSEA = 0.042), as shown in Table 3. This indicates that we could further examine the measurement invariance of the RTU and UTU MEFC on the other models that follow.

Measurement invariance was then evaluated using ΔCFI and ΔRMSEA between M_3_ (unconstrained model), M_4_ (measurement weights model), and M_5_ (structural weights model). In the model, M_3_ did not restrict any coefficients; M_4_ assumed that the indicator loadings for the corresponding construct in each group are the same; and M_5_ restricted the indicator loadings of the corresponding construct as well as the structural coefficients between the groups.

According to Table 3 ΔCFI was 0.001 between M_4_ and M_3_ and 0.002 between M_5_ and M_4_. The fact that all ΔCFI values are < 0.010 indicates that the models of M_1_, M_2_, M_3_, M_4_, and M_5_ have established measurement invariance across migration types. Between M_4_ and M_3_, ΔRMSEA was 0.001, and between M_5_ and M_4_, it was 0. The fact that all the ΔRMSEA values being <0.015 also indicates that the models M_1_, M_2_, M_3_, M_4_, and M_5_ in the RTU and UTU MEFC groups have established measurement invariance.

#### 3.2.2. Model fitness indices

**Figure 1:**
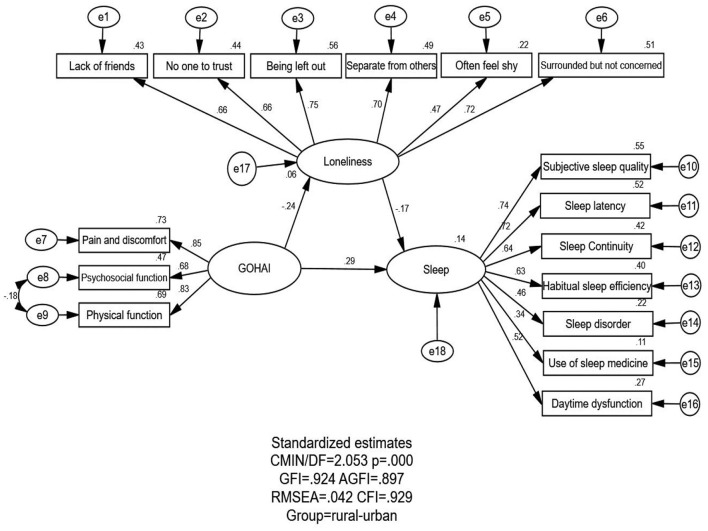
Structural equation modeling (SEM) analysis of the association between oral health status, loneliness, and sleep quality among the RTU MEFC (*n* = 525). All parameter estimates were statistically significant (*p* < 0.05). χ^2^, chi-square; GFI, goodness-of-fit index; AGFI, adjusted goodness-of-fit index; CFI, comparative fitness index; RMSEA, root mean square error of approximation.

**Figure 2:**
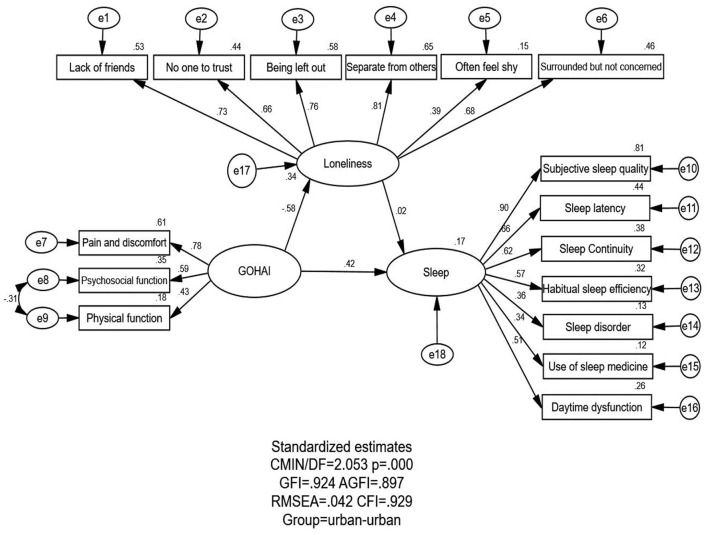
SEM analysis of the association between oral health status, loneliness, and sleep quality among the UTU MEFC (*n* = 88). The loneliness → sleep quality pathway was not significant, and the remaining parameter estimates were statistically significant (*p* < 0.05). χ^2^, chi square; GFI, goodness-of-fit index; AGFI, adjusted goodness-of-fit index; CFI, comparative fitness index; RMSEA, root mean square error of approximation.

Figures 1, 2, which show the proposed models for the RTU and UTU MEFC, respectively, contain three variables: oral health status, loneliness, and sleep quality. Table 3 displays the model fitness indices for various models, with M_1_ (RTU MEFC) and M_2_ (UTU MEFC) being the focus here. The RTU and UTU MEFC both had the same estimated value for model fitness: GFI = 0.924 > 0.90, AGFI = 0.897 > 0.80, CFI = 0.929 > 0.90, and RMSEA = 0.042 < 0.05, indicating that the theoretical model perfectly matched the empirical data for both RTU and UTU MEFC.

### 3.3. Assessment of the relationship between oral health status, loneliness, and sleep quality with SEM

#### 3.3.1. Association between oral health status and sleep quality

The association between oral health status, loneliness, and sleep quality is shown in Figures 1, 2 and Table 4.

**Table 4:** Standardized effects between GOHAI, loneliness, and sleep quality by migration type.

**Variable**	**Direct**		**Indirect**		**Total**	
	**RTU**	**UTU**	**RTU**	**UTU**	**RTU**	**UTU**
GOHAI → Sleep quality	0.292^**^	0.417^*^	0.042^***^	−0.010	0.334^**^	0.407^*^
Loneliness → Sleep quality	−0.174^**^	−0.018	–	–	−0.174^**^	−0.018
GOHAI → Loneliness	−0.242^**^	−0.582^**^	–	–	−0.242^**^	−0.582^**^

* p < 0.05,

** p < 0.01,

*** p < 0.001.

Oral health status and its indicators directly or indirectly affected sleep quality. Oral health status had a positive and direct effect on sleep quality among both RTU (standardized direct effects = 0.292) and UTU MEFC (standardized direct effects = 0.417). Oral health status could positively and indirectly affect sleep quality among the RTU MEFC (standardized indirect effects = 0.042). There was no indirect effect for the UTU MEFC. The positive effect of oral health status on sleep quality was stronger for the UTU MEFC than for the RTU MEFC (standardized total effects = 0.407 for the UTU MEFC; standardized total effects = 0.334 for the RTU MEFC). The standardized total effect was statistically significant for both groups.

#### 3.3.2. Association between oral health status and loneliness

Oral health status directly affected loneliness negatively for the RTU and UTU MEFC (standardized total effects = −0.242 for the RTU MEFC; standardized total effects = −0.582 for the UTU MEFC), indicating that better oral health among the MEFC correlated with less loneliness. A significant relationship was observed between oral health status and loneliness for the RTU and UTU MEFC; nevertheless, the association was higher in the UTU MEFC than in the RTU MEFC.

#### 3.3.3. Association between loneliness and sleep quality

Regarding the relationship between loneliness and sleep quality, loneliness among the RTU MEFC had a direct negative effect on sleep quality (standardized total effects = −0.174), indicating that a higher feeling of loneliness was associated with poor sleep quality. However, the relationship between loneliness and sleep quality among the UTU MEFC was not significant.

## 4. Discussion

### 4.1. Key study findings

This was the first study to explore the relationship between oral health status, loneliness, and sleep quality among the MEFC using SEM. We observed that oral health status was positively associated with sleep quality and negatively correlated with loneliness. In addition, loneliness was negatively associated with sleep quality. Significant differences were observed in the three aforementioned associations between the UTU and RTU MEFC.

### 4.2. Demographic characteristics of participants

Most RTU MEFC in this study were women. This might be because women take on more childcare responsibilities than men in traditional Chinese culture (33). Therefore, in this study, women accounted for a higher proportion of the MEFC who moved with their children to the city to care for their grandchildren than men. In addition, the UTU MEFC had a higher level of education, according to existing research findings, and demonstrated the persistent urban–rural divide in China (34). Most UTU MEFC received pensions, while fewer than half of the RTU MEFC received pensions, indicating that the government should increase the pension for the rural elderly (35). Most RTU MEFC migration included cross-district/county (followed by cross-prefecture-level and cross-provincial cities). In contrast, almost half of the UTU MEFC's migration was cross-district/county (followed by cross-provincial- and cross-prefecture-level cities). This may be because rural people have fewer resources than people from urban areas, which results in different migration space ranges (the RTU MEFC have a shorter migration space range than the UTU MEFC).

### 4.3. Status of MEFC's oral health, loneliness, and sleep quality

In this study, the MEFC had a higher GOHAI score compared to previous Chinese studies (36, 37), indicating better oral health. This may be because Weifang City is famous for its high vegetable production, and the intake of more vegetable fiber benefits oral health (38). Regarding the migration type difference, the UTU MEFC had better oral health than the RTU MEFC, in accordance with previous findings among older people in the USA (39), Indonesia (40), and Brazil (41). The reason may be that older rural people use less oral health services and have poor hygiene practices (42).

In this study, the UTU and RTU MEFC had fairly low loneliness, which differed from existing studies in the USA (43), Korea (44), and the Philippines (45) that revealed loneliness to be higher in the migrant elderly. A reason may be that accompanying family members in the inflow city decrease loneliness among the MEFC. Moreover, the RTU MEFC had longer sleep latency and used sleep medications more frequently than the UTU MEFC. This is consistent with previous studies on poor sleep quality among older Chinese people in rural areas (46, 47).

### 4.4. The relationship between oral health status, loneliness, and sleep quality

#### 4.4.1. Oral health status and sleep quality

This study uncovered a positive association between oral health status and sleep quality among the MEFC, in accordance with the results of studies in the Korean (48) and Italian (49) elderly. The results also explained why people with poor oral health had poor sleep quality. Wang et al. observed that oral health was strongly associated with sleep and a major determinant of sleep quality among the Chinese elderly (50). Yuan et al. also revealed that sleep quality was worse in patients with periodontitis than in those without periodontitis among elderly Chinese dental patients, with patients with severe periodontitis having the worst sleep quality (51). Previous studies explored the effects of cognition (52), depression (53), and chronic diseases (54, 55) on sleep quality among the elderly. Yet, few studies have clarified the influence of oral health on sleep quality among the migrant elderly. Therefore, this study extends beyond previous studies.

Concerning the migration type difference, the relationship between oral health status and sleep quality was stronger in the UTU MEFC than in the RTU MEFC. A reason can be that the UTU MEFC may be more health literate (56) and have a healthier lifestyle and more focus on their health than the RTU MEFC, which may further result in a higher correlation between oral health status and sleep quality. Moreover, for the RTU MEFC, due to their migration from rural areas, the new urban environment, interpersonal relationships, and living habits may have a greater impact on them, which may lead to the neglect of their oral health and less attention paid to sleep quality.

#### 4.4.2. Loneliness and sleep quality

Loneliness was negatively correlated with sleep quality among the MEFC in this study, consistent with Griffin et al.'s study, which showed that higher levels of loneliness were associated with more sleep disturbance among older Americans (57). Fu et al.'s study of older people in Shandong Province also revealed that subjective sleep quality, time to sleep, sleep disturbance, and daytime dysfunction were significantly associated with loneliness (58).

Regarding the migration type difference, loneliness had a negative association with sleep quality for the RTU MEFC. However, no such association was observed for UTU MEFC in contrast to Cao's study in China (59), which revealed that loneliness was significantly associated with sleep quality among the retired elderly in cities. A reason might be that the movement of RTU MEFC from a rural area resulted in more environmental or lifestyle differences between the inflow city and their hometown. Thus, they are more anxious and lonelier, resulting in worse sleep quality. However, the differences between the inflow city and their hometowns might be less for the UTU MEFC, resulting in no empirical relationship between loneliness and sleep quality.

#### 4.4.3. Oral health status and loneliness

There was a negative association between oral health status and loneliness among the MEFC in this study, similar to Hajek et al.'s study (60), which revealed that previous studies had confirmed a relationship between oral health and loneliness. Ma's study of older people in the Chinese community revealed that chewing function, swallowing function, tooth loss, dental function, and toothache were factors influencing loneliness and that attention should be paid to maintaining and promoting oral function and reducing loneliness among the elderly (61).

As for the disparity in migration type, the negative effect of oral health status on loneliness was stronger for the UTU MEFC than for the RTU MEFC. The reason might be that the UTU MEFC enjoyed medical insurance and better oral health services in their hometown (62). In addition, their comparatively better lifestyle may result in better oral health and fewer oral problems. However, when oral health challenges occur, the disconnection of medical insurance after migration from their hometown to the inflow city, and their unfamiliarity with the medical facilities and medical services, make them feel lonely. In contrast, the hometown lifestyle of RTU MEFC may not be as good as that of the UTU MEFC, resulting in more oral problems to which they would have adapted, even when the oral problems occurring in the inflow city may still cause fewer mental fluctuations and loneliness.

### 4.5. Future implications

As a large group in the urbanization process, more attention should be paid to the health status of the MEFC. Due to the huge urban–rural disparity in China, different supportive measures need to be taken for the UTU and RTU MEFC. First, health education on oral health, loneliness, and sleep should be provided to the MEFC, especially the RTU MEFC. Second, the government should increase the pension of the RTU MEFC groups to reduce the urban–rural disparity. Third, because oral health status was positively correlated with sleep quality and negatively correlated with loneliness, relevant policies (i.e., increasing the reimbursement ratio of dental services) are needed to improve the oral health status of the MEFC group. Fourth, as a negative relationship between loneliness and sleep quality only existed in the RTU MEFC group in this study, it is suggested that the community pay more attention to the RTU MEFC (such as by organizing more community activities and visiting them more often). At the same time, family members should provide more care to them to reduce their loneliness and help them adapt to a new life in the inflow city.

### 4.6. Limitations

This study had several limitations. First, this was a cross-sectional study, and causality could not be predicted. Second, the study was conducted using a self-assessment scale, which might have introduced some bias. Third, other confounding factors that might also affect sleep quality among the MEFC were not included in this study (such as stress and depression) and should be included in future studies. Fourth, this study could not be conducted in other places, such as Shanghai, due to the epidemic. Therefore, no further areas could be validated.

## 5. Conclusions

This study clarified sleep quality among the MEFC and an empirical relationship between oral health status, loneliness, and sleep quality among the MEFC in Weifang, Shandong Province. The total sleep quality score of the MEFC was 4.47 ± 3.60, which was higher than that in previous studies. Oral health and sleep quality were significantly and positively correlated for the RTU and UTU MEFC, with a slightly stronger association for the UTU MEFC. There was a significant negative correlation between oral health and loneliness among the RTU and UTU MEFC, with a stronger correlation in the UTU MEFC. Therefore, loneliness could negatively impact sleep quality among the RTU MEFC. However, no statistical association was observed in the UTU MEFC.

## Data availability statement

The raw data supporting the conclusions of this article will be made available by the authors, without undue reservation.

## Ethics statement

The studies involving human participants were reviewed and approved by Ethical Committee of Shandong University (No. 20180225). The patients/participants provided their written informed consent to participate in this study.

## Author contributions

Conceptualization, writing—review and editing, and supervision: SL, LX, FK, and XG. Methodology, validation, resources, and data curation: FK. Software, formal analysis, and writing—original draft preparation: XJ. Investigation: XJ, GL, JX, HL, JW, and MP. The published version of the manuscript has been approved by all authors who have read it.
